# The acute effects of different levels of intermittent negative pressure on peripheral circulation in patients with peripheral artery disease

**DOI:** 10.14814/phy2.14241

**Published:** 2019-10-20

**Authors:** Henrik Hoel, Lars Øivind Høiseth, Gunnar Sandbæk, Jon Otto Sundhagen, Iacob Mathiesen, Jonny Hisdal

**Affiliations:** ^1^ Institute of Clinical Medicine Faculty of Medicine University of Oslo Oslo Norway; ^2^ Department of Vascular Surgery Oslo University Hospital Oslo Norway; ^3^ Otivio AS Oslo Norway; ^4^ Department of Anesthesiology Oslo University Hospital Oslo Norway; ^5^ Department of Radiology Oslo University Hospital Oslo Norway

**Keywords:** Arterial blood flow, intermittent negative pressure, peripheral artery disease, skin blood flow

## Abstract

Intermittent negative pressure (INP) applied to the lower leg induces acute increase in arterial and skin blood flow. The aim of this study was to identify the optimal level of INP to increase blood flow in patients with lower extremity peripheral artery disease (PAD). We investigated the acute effects of different levels of INP in 16 subjects (7 women and 9 men, mean (SD) age 71(8) years) diagnosed with PAD. During application of INP in a pressure chamber sealed below the knee, arterial blood flow was continuously recorded in the dorsalis pedis artery or tibialis posterior artery (ultrasound Doppler), and skin blood flow was continuously recorded at the pulp of the first toe (laser Doppler). Different pressure levels (0, −10, −20, −40, and −60 mmHg) were tested in randomized order. Maximal arterial blood flow relative to baseline (median [25th, 75th percentiles]) was: 0 mmHg; 1.08 (1.02, 1.13), −10 mmHg; 1.11 (1.07, 1.17), −20 mmHg; 1.18 (1.11, 1.32), −40 mmHg; 1.39 (1.27, 1.91) and −60 mmHg; 1.48 (1.37, 1.78). Maximal laser Doppler flux (LDF) relative to baseline was: 0 mmHg; 1.06 (1.02, 1.12), −10 mmHg; 1.08 (1.05, 1.16) −20 mmHg; 1.12 (1.06, 1.27), −40 mmHg; 1.24 (1.14, 1.50) and −60 mmHg; 1.35 (1.10, 1.70). There were significantly higher maximal arterial blood flow and maximal LDF at −40 mmHg compared with −10 mmHg (*P* = 0.001 and *P* = 0.025, respectively). There were no significant differences in maximal arterial blood flow and maximal LDF between 0 and −10 mmHg (both *P* = 1.0), or between −40 and −60 mmHg (both *P* = 1.0). INP of −40 mmHg was the lowest negative pressure level that increased blood flow.

## Introduction

Peripheral artery disease (PAD) comprise all conditions resulting in obstruction of blood flow in arteries, exclusive of the coronary, and intracranial vessels (Ouriel 2001). Atherosclerosis leading to obstruction of blood flow to the lower extremities is a common manifestation of PAD. Lower extremity PAD may lead to ischemia causing pain while walking that is relieved by rest, termed intermittent claudication. This may progress to critical limb ischemia resulting in pain at rest, tissue loss, and gangrene (Norgren et al. 2007).

Standard treatment for PAD includes smoking cessation, pharmacological therapy with antiplatelet agents and statins, and supervised physical exercise (Conte et al. 2015). Supervised exercise programs are recommended to improve functional status and reduce leg symptoms and should be discussed as a treatment option for patients with intermittent claudication before revascularization (Gerhard‐Herman et al. 2017). However, a systematic review from 2016 reported that only 1 in 3 patients with intermittent claudication were suitable for and willing to participate in supervised exercise programs (Harwood et al. 2016). For patients with severe PAD, endovascular or open surgery may be options to achieve revascularization, but the ability to perform endovascular or open surgery depends on the localization and extent of the disease. A significant proportion of the patients also have severe co‐morbidities (Ouriel 2001), which may contraindicate surgery.

The effects of intermittent negative pressure (INP) to improve blood flow in patients with PAD have been investigated since the early 20th century (Sinkowitz and Gottlieb 1917; Landis and Gibbon 1933; Herrmann and Reid 1934; Herrmann 1935). A number of studies have described different devices applying INP by alternately removing air from and venting a pressure chamber sealed around the patients’ leg or lower body (Sinkowitz and Gottlieb 1917; Landis and Gibbon 1933; Herrmann and Reid 1934; Smyth 1969; Himmelstrup et al. 1991; Sundby et al. 2017). In 1969, Smyth et al. applied intermittent negative pressure to the lower limbs of patients with different stages of PAD and observed improvement in resting and post ischemic blood flow and walking distance after 6 weeks of treatment (Smyth, 1969). A Danish study from 1983 showed that locally applied constant negative pressure increased vascular resistance and reduced subcutaneous blood flow, but the vasoconstriction was abolished by local nervous blockade induced by low doses of lidocaine injected subcutaneously, suggesting that the vasoconstriction was due to a local sympathetic veno‐arterial axon reflex mechanism, which constricts the arterioles when veins are distended (Skagen and Henriksen 1983). One study has demonstrated that INP combined with heated water applied to the arm was effective to prevent hypothermia in patients undergoing laparotomy (Rein et al. 2007). In this study, it was suggested that the observed increase in blood flow was due to increased pressure difference between the arterial and venous system, and by avoidance of the veno‐arterial reflex. A recent study on healthy volunteers from our research group showed that 2 min of constant negative pressure applied to the lower extremities decreased blood velocity in the dorsalis pedis artery (ADP) or tibialis posterior artery (ATP), and skin blood flow (Sundby et al. 2016). The same study demonstrated that INP of −40 mmHg applied to the lower extremities increased maximal arterial blood velocity 44% (95% CI 33–55) above baseline. In another study using the same experimental setup on patients with PAD, maximal arterial blood velocity increased 46% (95% CI 36–57) above baseline (Sundby et al. 2017). This effect thus seems comparable between healthy volunteers and patients with PAD. The three latter studies reported INP levels of −40 mmHg, in cycles of 10 sec negative pressure, and 7 sec atmospheric pressure to be effective to increase arterial and skin blood flow (Rein et al. 2007; Sundby et al. 2016; 2017).

Although several studies have demonstrated increased blood flow in the extremity during application of INP, the optimal level of INP to improve blood flow is unknown. Our hypothesis was that blood flow in the foot increases with increasing magnitude of INP until a certain level. Hence, the aim of the present study was to identify the optimal level of INP applied in sequences of 10 sec negative pressure and 7 sec atmospheric pressure to increase blood flow in the lower extremities in subjects with PAD.

## Methods

### Participants

Study subjects were recruited from the out‐patient clinic at the Department of Vascular Surgery, Oslo University Hospital, Oslo, Norway. Subjects with resting ankle‐brachial index (ABI) <0.9 and symptomatic claudication or radiological detected PAD were included. Subjects undergoing recent (less than three months) endovascular or open surgical revascularization were considered not eligible for the study.

### Experimental setup and measurements

We registered age, sex, weight, height, comorbidities, smoking status, medications, main localization of the disease, previous revascularization, and patient reported maximal walking distance for all subjects based on a questionnaire and the subjects’ medical record at Oslo University Hospital.

All subjects were encouraged not to eat, and to refrain from tobacco and caffeine two hours before the experiments. Measurements were conducted in a temperature stable environment of 22–24°C. The subjects´ most symptomatic leg was chosen as the test leg.

ABI was measured after 5 min of rest, with the subject in supine position using a continuous wave 8 MHz Doppler probe (Macrolab, STR Teknikk, Aalesund, Norway), in accordance with the guidelines from the American Heart Association (Aboyans et al. 2012).

Pulse volume recording (PVR) amplitude was measured with an air‐plethysmography cuff (Macrolab, STR Teknikk, Aalesund, Norway) placed at the lower leg above the malleoli. Normal arterial inflow to the extremity is pulsatile, leading to measurable changes in lower limb volume within each cardiac cycle (Hashimoto et al. 2016), and in the case of PAD, the waveform of PVR becomes dampened.

We measured the peak systolic velocity and the diameter of the ADP or ATP using a triplex ultrasound scanner (GE LOGIQ 9 Ultrasound, Wauwatosa, Wisconsin, USA). A pulsed 10 MHz Doppler probe (SD‐50, GE Vingmed Ultrasound, Horten, Norway) was used to measure the arterial blood velocity during application of INP. The probe was fixed to the foot with surgical tape above the ATP or ADP, at the place where the best Doppler signal was achieved. Based on the peak systolic velocity measured with the ultrasound triplex scan, the Doppler ultrasound was calibrated to record the exact arterial blood velocity in the ATP or ADP.

A laser Doppler flow meter was used to monitor microvascular blood perfusion in acral skin using a laser Doppler probe attached to the pulp of the first toe (PeriFlux 5000, Perimed AB, Jarfalla, Sweden). The same probe recorded skin temperature.

Pressure inside the pressure chamber was recorded by a digital manometer (Macrolab, STR Teknikk, Aalesund, Norway).

Systemic blood pressure was measured non‐invasively beat‐by‐beat, by a Finometer (FMS, Finapres medical systems BV, Arnhem, Netherlands) attached to the third finger of the right arm.

**Figure 1:**
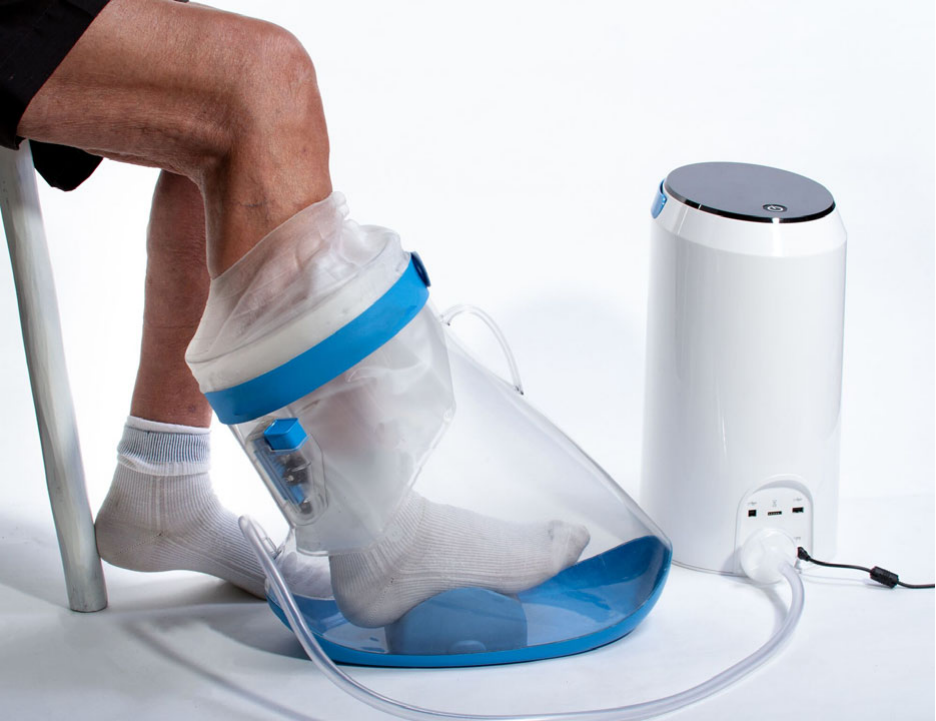
Intermittent negative pressure generated in a pressure chamber sealed around the lower leg, by control unit that is actively removing air from and passively venting the pressure chamber.

With the subject sitting in a chair, all probes were connected, and the foot was carefully introduced into the pressure chamber. The foot arch was placed on a positioner to avoid the front foot and the heel to touch the pressure chamber. The pressure chamber was sealed just below the knee using a customized thermoplastic elastomer seal and coupled by air hoses to a control unit (FlowOx, Otivio AS, Oslo, Norway) that generated INP by actively removing air from and passively venting the pressure chamber (Fig. 1). The control unit was programmed to apply time sequences of 10 sec of negative pressure and 7 sec of atmospheric pressure during all tests. Before the start of the experiments, adequate signals were confirmed. The subjects were encouraged to sit relaxed with approximately 130° flexion in the knee joint during the experiment. For all the subjects, experimental data were first sampled in a 5‐min sequence at atmospheric pressure, before INP sequences, each lasting 5 min were sampled. Pressure levels of −10, −20, −40 and −60 mmHg were tested with a 5‐min wash‐out period between the tests. To account for possible carry‐over effects between the pressure levels, the test order of the INP levels was randomized using an online randomization software (Research Randomizer, www.randomizer.org).

Data recorded during the tests were sampled at 300 Hz, and averaged beat‐by‐beat, gated by the R‐waves of a three lead ECG, using custom‐made software (REGIST3, Morten Eriksen, University of Oslo, Norway). The software calculated beat‐by‐beat blood flow in the ADP or ATP during the tests by adding information on the angle of insonation and the vessel diameter measured with the ultrasound triplex scan before application of INP. The beat‐by‐beat data were resampled to 2 Hz for further analyses. Obviously erroneous data due to for example motion artefacts were removed from the dataset, giving 15 complete INP cycles, each lasting 17 sec, for each subject at each pressure level.

### Statistical analyses

Descriptive statistics are presented as mean (standard deviation [SD]) or median (25th, 75th percentile). Shapiro–Wilks tests were performed to assess if the flow and LDF data were normally distributed at every 0.5 sec of the INP cycles, giving *P* < 0.001 for all tests. Therefore, these data were not assumed to have a normal distribution.

For each subject and INP level, median arterial blood flow, laser Doppler flux (LDF), and mean arterial pressure (MAP) every 0.5 sec of the 17 sec cycle of INP were divided by the median value at time 0 (baseline) for the pressure level being tested, giving an intra‐subject relative median value for each 0.5 sec of the 17 sec INP cycle. The aggregated medians of flow and LDF for all subjects were plotted to illustrate the differences between the pressure levels. From the intra‐subject median values, the maximal values were found for each INP level, giving the maximal blood flow and LDF relative to baseline for that subject and INP level. We used Friedman test for non‐normally distributed data to examine the overall null hypothesis of no significant differences in any of the rank sums of maximal blood flow, LDF and mean arterial pressure (MAP) between the INP levels (Eisinga et al. 2017). For pairwise comparisons of maximal blood flow and LDF between the INP levels, Dunn’s post hoc tests were performed with Bonferroni adjustment for multiple comparisons. We used SPSS (IBM Statistics for Windows, Version 25.0. IBM Corp., Armonk, NY, USA) and SigmaPlot (SigmaPlot, Version 12.0, Systat Software Inc., San Jose, CA, USA) for statistical analyses and plotting of data.

A recent study on patients with PAD reported a mean (SD) blood velocity in ADP of 6.7 (3.3) cm/sec (Sundby et al. 2017). To detect an increase in blood velocity of 40% during INP, at least 12 subjects must be included in the current study given a significance level of 0.05 and a power of 80%.

### Ethics

The project was approved by the Regional Committee for Medical and Health Research Ethics in Norway (ref: 2014/1967) and registered at ClinicalTrials.gov (ref: SD0321063). Written informed consent was obtained from all subjects before the start of the experiments.

## Results

Sixteen subjects with PAD Fontaine stage I (1 subject) and Fontaine stage II (15 subjects) were included in the study (Table 1). Patient reported maximal walking distance was 225 (100, 500) meters and ABI was 0.62 (0.15). Twelve subjects had femoropopliteal disease, two had aortoiliac disease, and two had infrapopliteal disease. Six subjects had previously undergone revascularization of the tested leg.

**Table 1:** Subject´s characteristics, *n* = 16.

Age, years*	71 (8)
Male sex	9 (56)
Body mass index, kg/m^2^ *	23.5 (3.1)
Test leg right	11 (69)
Symptomatic bilateral PAD	9 (56)
Ankle‐brachial index test leg*	0.62 (0.15)
Pulse volume recording test leg, mm*	8.3 (3.4)
Blood flow measured in dorsalis pedis artery	12 (75)
Smoking status, current smoker/ex‐smoker/nonsmoker	3 (19)/8 (50)/5 (31)
Diabetes mellitus	1 (6)
Chronic renal failure	1 (6)
Hypercholesterolemia	9 (56)
Hypertension	10 (63)
Coronary artery disease	8 (50)
Cerebrovascular disease	2 (13)
Antiplatelet agents	14 (88)
Lipid lowering agents	15 (94)
Antihypertensive agents	11 (69)
Patient reported maximal walking distance, meters†	225 (100, 500)
Fontaine stage	
I	1 (6)
IIa	9 (56)
IIb	6 (38)
III	0 (0)
IV	0 (0)
Main localization of disease	
Aortoilliac	2 (13)
Feomorpoliteal	12 (75)
Infrapopliteal	2 (13)
Previous revascularization of test leg	
Endovascular	5 (31)
Open surgery	1 (6)

Values are number (%) unless otherwise stated.

* Mean (standard deviation).

^†^ Median (25th, 75th percentile).

**Figure 2:**
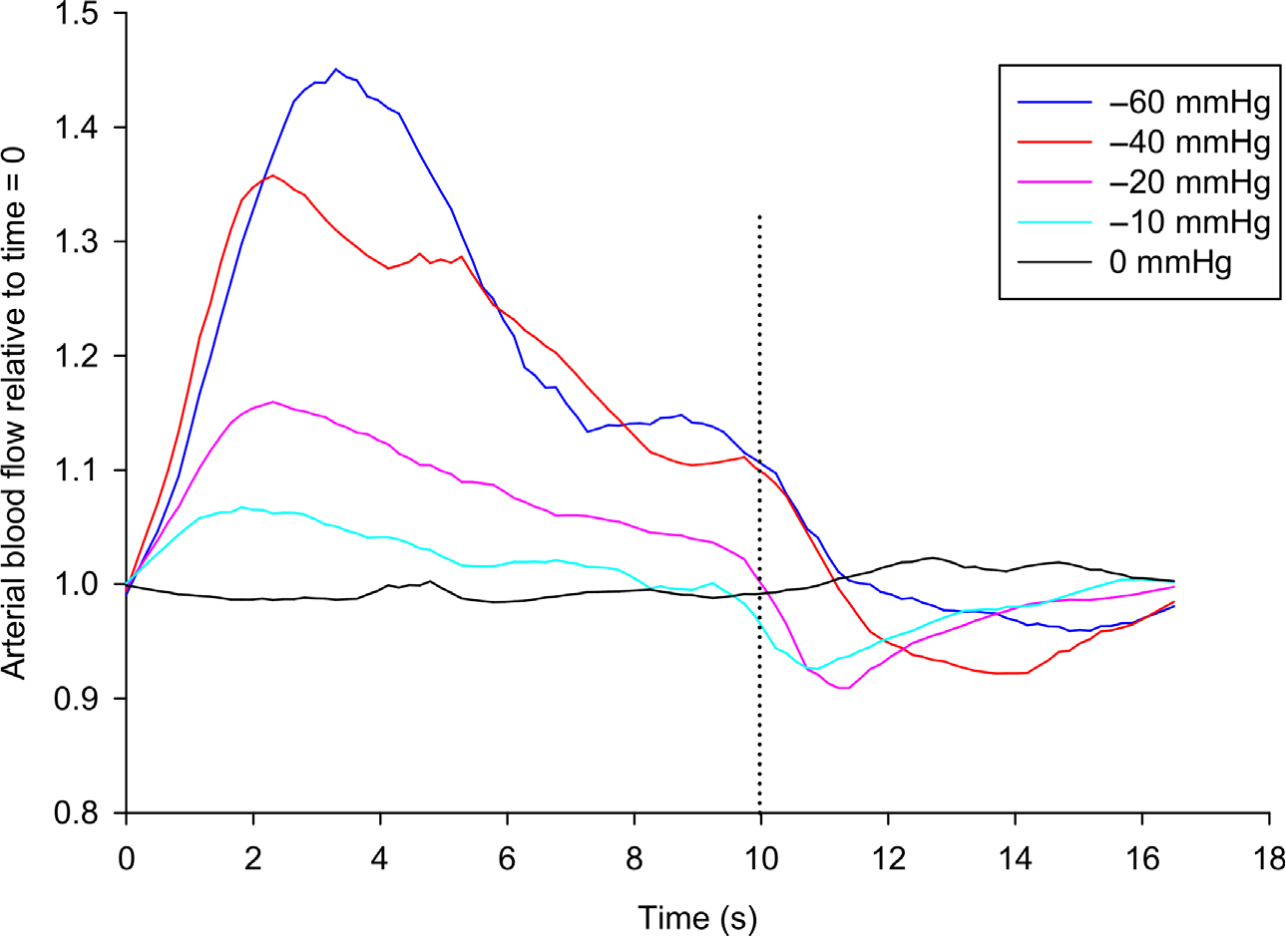
Arterial blood flow in the foot during the 17 sec cycles of intermittent negative pressure. Aggregated medians relative to baseline (time = 0 sec) plotted every 0.5 sec for all patients (*n* = 16) at each pressure level. Dashed line indicates switch from negative pressure to atmospheric pressure.

Maximal blood flow during the 17 sec cycles at pressure levels of −60 and −40 mmHg were reached after 3 sec and 2 sec, respectively, followed by a gradual decrease in flow until 10 sec, when the negative pressure was turned off. During the 7 sec with atmospheric pressure, the blood flow decreased below baseline, before returning to baseline after 17 sec (Fig. 2).

**Figure 3:**
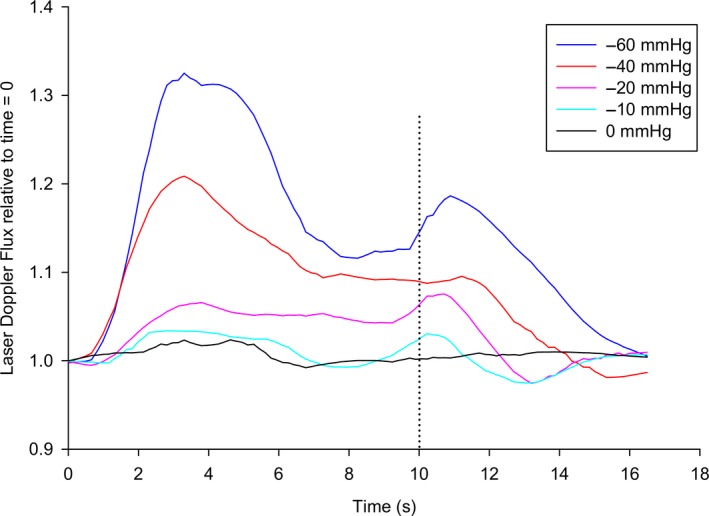
Laser Doppler Flux in acral skin of the foot during the 17 sec cycles of intermittent negative pressure. Aggregated medians relative to baseline (time = 0 sec) plotted every 0.5 sec for all patients (*n* = 16) at each pressure level. Dashed line indicates switch from negative pressure to atmospheric pressure.

Maximal LDF during the 17 sec cycles at pressure levels of −60 and −40 mmHg were reached after 3 sec, followed by a gradual decrease in LDF until the negative pressure was turned off after 10 sec. During the last 7 sec with atmospheric pressure, we observed a slight increase in LDF before returning to baseline after 17 sec (Fig. 3).

**Figure 4:**
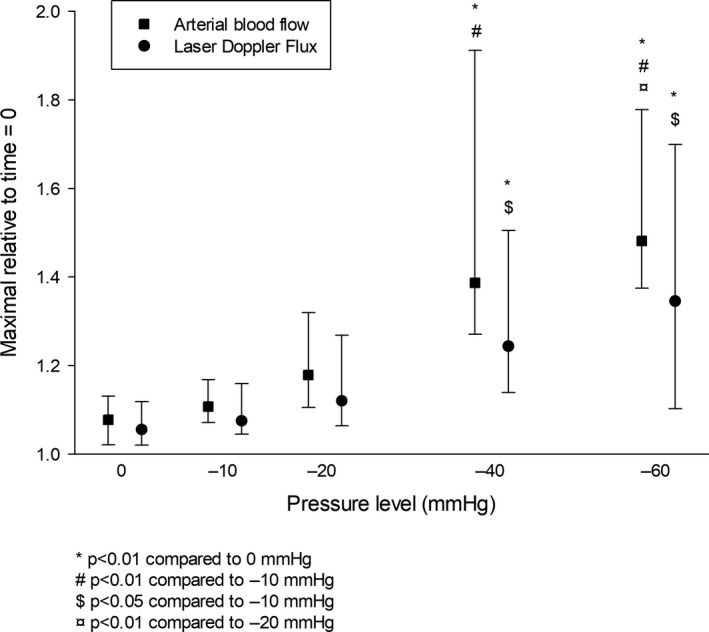
Maximal arterial blood flow and Laser Doppler Flux during the 17 sec cycles of intermittent negative pressure. Medians and 25th–75th percentiles for all patients (*n* = 16) relative to baseline (time = 0 sec) at different pressure levels.

Maximal arterial blood flow for each pressure level, relative to baseline was: 0 mmHg; 1.08 (1.02, 1.13), −10 mmHg; 1.11 (1.07, 1.17), −20 mmHg; 1.18 (1.11, 1.32), −40 mmHg; 1.39 (1.27, 1.91) and −60 mmHg; 1.48 (1.37, 1.78). Maximal LDF for each pressure level relative to baseline was: 0 mmHg; 1.06 (1.02, 1.12), −10 mmHg; 1.08 (1.05, 1.16), −20 mmHg; 1.12 (1.06, 1.27), −40 mmHg; 1.24 (1.14, 1.50) and −60 mmHg; 1.35 (1.10, 1.70) (Fig. 4).

Overall, there were significant differences in maximal arterial blood flow and maximal LDF between the pressure levels (both *P* < 0.001). For pairwise comparisons of maximal arterial blood flow, there were significant differences between: 0 and −40 mmHg (*P* < 0.001), 0 and −60 mmHg (*P* < 0.001), −10 and −40 mmHg (*P* = 0.001), −10 and −60 mmHg (*P* < 0.001) and −20 and −60 mmHg (*P* = 0.005). For pairwise comparisons of maximal LDF, there were significant differences between: 0 and −40 mmHg (*P* = 0.001), 0 and −60 mmHg (*P* < 0.001), −10 and −40 mmHg (*P* = 0.025) and ‐10 mmHg and ‐60 mmHg (*P* = 0.012). There were no significant differences in maximal arterial blood flow or maximal LDF between 0 and −10 mmHg (both *P* = 1.0) or between −40 and −60 mmHg (both *P* = 1.0). There was no significant difference in the maximal change of MAP between the pressure levels (*P* = 0.434).

## Discussion

The main finding of the present study was that application of INP to the lower extremity with pressure levels of −40 mmHg and −60 mmHg increased maximal arterial blood flow and skin blood flow in the foot compared to 0 and −10 mmHg in subjects with PAD. There were no significant differences in maximal arterial blood flow and skin blood flow between −60 and −40 mmHg. Hence, −40 mmHg was the lowest level of negative pressure that induced such changes. An INP level of ‐10 mmHg did not significantly induce an acute increase in maximal arterial blood flow and skin blood flow compared with atmospheric pressure alone.

The finding that an INP level of −40 mmHg induced acute increase in arterial blood flow and skin blood flow is in line with previous studies (Sundby et al. 2016; Sundby et al. 2017; 2018). However, the mechanisms of action leading to increased blood flow during INP are not well described in the literature. According to Poiseuille’s law for laminar flow through a cylindric tube, the flow is dependent on the pressure difference between the two sides of the tube, the radius of the tube, and the viscosity of the liquid flowing through the tube (Pfitzner 1976). INP may affect both the pressure differences between the arterial and venous side of the capillary bed, as well as the vessel diameter. One previous study observed an increased blood flow also in a 5‐min period after INP treatment was ended (Sundby et al. 2017). This suggests that the leg benefits from the treatment for a longer time than just during the period of INP. This may be explained by the increased blood flow during INP leading to increased shear stress between the blood and the endothelium of the arterial wall, and thereby inducing flow‐mediated vasodilatation (Joannides et al. 1995). Hence, flow‐mediated vasodilatation may be one physiological explanation for the potential clinical benefits of INP treatment for patients with PAD that have been presented in a number of studies (Herrmann and Reid, (1934); Sundby et al. 2017; Himmelstrup et al. 1991; Smyth 1969; Mehlsen et al. 1993).

The theoretical rationale of applying INP instead of constant negative pressure is to avoid the veno‐arterial reflex mechanism that induces vasoconstriction on the arterial side when veins become distended (Skagen and Henriksen 1983). Activation of this reflex is probably dependent both on the level of negative pressure applied and the length of the negative pressure periods and atmospheric pressure periods. If the negative pressure periods are shortened or the atmospheric pressure periods are lengthened, there is a possibility that a higher pressure difference (higher INP level) will induce an even greater increase in blood flow. Previous studies of INP treatment of patients with PAD have tested pressure levels of ‐120, −150, and −200 mmHg (Landis and Gibbon 1933; Smyth 1969; Gill and Walder 1974; Himmelstrup et al. 1991; Mehlsen et al. 1993), but the way in which negative pressure was applied, and the length of negative pressure periods and atmospheric pressure periods varies between the different studies.

Sundby et al. assessed pain during application of INP in patients with PAD, finding a mean verbal numerical rating pain scale of 0 at an INP level of −40 mmHg (Sundby et al. 2017). Even though the potential side effects of INP treatment are few and the discomfort for the patients is little, it is reasonable to not expose patients to local pressure changes that are higher than what is necessary to achieve optimal blood flow.

The level of evidence of the clinical effects of INP treatment in the literature is scarce and should be a subject for further research. In this study, we demonstrated that a pressure level of −10 mmHg did not significantly increase arterial blood flow and skin blood flow. This might thus be the INP level of a sham device in a randomized sham‐controlled trial designed to explore if INP can contribute in the treatment of patients with lower extremity PAD.

There are some limitations in this study. It was not possible to measure the vessel diameters in the pressure chamber during INP. Therefore, the diameters of ADP and ATP were measured before application of INP, and arterial blood velocity was measured during application of INP. Hence, the flow calculations were based upon the vessel diameter before application of INP. If application of INP leads to a shear stress induced vasodilatation, this may have led to underestimation of arterial blood flow. We used a pulsed 10 MHz ultrasound Doppler probe fixed to the subjects’ feet over the ADP or ATP to monitor blood flow during the application of INP. Small displacements of the probe caused by the subject changing the position of the leg during the experiment may decrease the quality of the Doppler signal, leading to periods of underestimation of blood flow. Furthermore, this study was conducted in a standardized environment on subjects with PAD, and one should be careful with generalization of the results to other patient groups or settings.

This study concludes that INP treatment of subjects with PAD with pressure levels of −40 and −60 mmHg applied in cycles of 10 sec of negative pressure and 7 sec of atmospheric pressure induced acute increase in arterial and skin blood flow. INP of −40 mmHg was the lowest negative pressure level that increased blood flow.

## Conflict of Interest

HH is employed by Otivio with funding from the Norwegian Research Council (NFR grant no: 285758). IM is the CSO, co‐founder and a shareholder in Otivio AS. Otivio AS has the commercial rights to the INP technology (FlowOx) used in the study. None of the other authors have any conflicts of interest, financial, or otherwise. The authors alone are responsible for the content and writing of the paper.

## Supporting information




**Table S1** **.** Pairwise comparisons of the different levels of intermittent negative pressure.

## Data Availability

All data supporting the results in this paper will be publicly available on the repository Figshare.
