# Supplementary material for: The acute effects of different levels of intermittent negative pressure on peripheral circulation in patients with peripheral artery disease
Source: Physiol Rep. 2019 Oct 20;7(20):e14241. doi: 10.14814/phy2.14241 (PMC6801220; doi:10.14814/phy2.14241)
Supplement: Supplementary file 1 — Table S1 . Pairwise comparisons of the different levels of intermittent negative pressure. [file PHY2-7-e14241-s001.docx]

**S1 Table Pairwise comparisons of the different levels of intermittent negative pressure.**

Friedman tests were carried out to compare the maximal arterial blood flow and maximal laser Doppler flux between the pressure levels (both p<0.001). Dunn-Bonferroni post hoc tests were carried out for pairwise comparisons.

| Maximal arterial blood flow | | | | | Maximal laser Doppler flux | | | | |  |
| --- | --- | --- | --- | --- | --- | --- | --- | --- | --- | --- |
| Sample 1 vs Sample 2 | Test Statistic | Std. Error | Std. Test Statistic | p-value | Adjusted  p-value # | Test Statistic | Std. Error | Std. Test Statistic | p-value | Adjusted  p-value # |
| 0 mmHg vs -10 mmHg | -0.375 | 0.559 | -0.671 | 0.502 | 1.000 | -0.562 | 0.559 | -1.006 | 0.314 | 1.000 |
| 0 mmHg vs -20 mmHg | -1.312 | 0.559 | -2.348 | 0.019 | 0.189 | -1.062 | 0.559 | -1.901 | 0.057 | 0.573 |
| 0 mmHg vs -40 mmHg | -2.562 | 0.559 | -4.584 | <0.001 | <0.001 | -2.250 | 0.559 | -4.025 | <0.001 | 0.001 |
| 0 mmHg vs -60 mmHg | -3.250 | 0.559 | -5.814 | <0.001 | <0.001 | -2.375 | 0.559 | -4.249 | <0.001 | <0.001 |
| -10 mmHg vs -20 mmHg | -0.938 | 0.559 | -1.677 | 0.094 | 0.935 | -0.500 | 0.559 | -0.894 | 0.371 | 1.000 |
| -10 mmHg vs -40 mmHg | -2.188 | 0.559 | -3.913 | <0.001 | 0.001 | -1.688 | 0.559 | -3.019 | 0.003 | 0.025 |
| -10 mmHg vs -60 mmHg | -2.875 | 0.559 | -5.143 | <0.001 | <0.001 | -1,812 | 0.559 | -3.242 | 0.001 | 0.012 |
| -20 mmHg vs -40 mmHg | -1.250 | 0.559 | -2.236 | 0.025 | 0.253 | -1.188 | 0.559 | -2.124 | 0.034 | 0.336 |
| -20 mmHg vs -60 mmHg | -1.938 | 0.559 | -3.466 | 0.001 | 0.005 | -1.312 | 0.559 | -2.348 | 0.019 | 0.189 |
| -40 mmHg vs -60 mmHg | -0.688 | 0.559 | -1.230 | 0.219 | 1.000 | -0.125 | 0.559 | -0.224 | 0.823 | 1.000 |

# Significance values have been adjusted by the Bonferroni correction for multiple tests.
